# Racism, Gun Ownership and Gun Control: Biased Attitudes in US Whites May Influence Policy Decisions

**DOI:** 10.1371/journal.pone.0077552

**Published:** 2013-10-31

**Authors:** Kerry O’Brien, Walter Forrest, Dermot Lynott, Michael Daly

**Affiliations:** 1 Behavioural Studies, Monash University, Melbourne, Australia; 2 School of Psychological Sciences, University of Manchester, Manchester, United Kingdom; 3 Department of Psychology, Lancaster University, Lancaster, United Kingdom; 4 Institute of Sociomanagement, University of Stirling, Stirling, Scotland, United Kingdom; University of Queensland, Australia

## Abstract

**Objective:**

Racism is related to policies preferences and behaviors that adversely affect blacks and appear related to a fear of blacks (e.g., increased policing, death penalty). This study examined whether racism is also related to gun ownership and opposition to gun controls in US whites.

**Method:**

The most recent data from the American National Election Study, a large representative US sample, was used to test relationships between racism, gun ownership, and opposition to gun control in US whites. Explanatory variables known to be related to gun ownership and gun control opposition (i.e., age, gender, education, income, conservatism, anti-government sentiment, southern vs. other states, political identification) were entered in logistic regression models, along with measures of racism, and the stereotype of blacks as violent. Outcome variables included; having a gun in the home, opposition to bans on handguns in the home, support for permits to carry concealed handguns.

**Results:**

After accounting for all explanatory variables, logistic regressions found that for each 1 point increase in symbolic racism there was a 50% increase in the odds of having a gun at home. After also accounting for having a gun in the home, there was still a 28% increase in support for permits to carry concealed handguns, for each one point increase in symbolic racism. The relationship between symbolic racism and opposition to banning handguns in the home (OR1.27 CI 1.03,1.58) was reduced to non-significant after accounting for having a gun in the home (OR1.17 CI.94,1.46), which likely represents self-interest in retaining property (guns).

**Conclusions:**

Symbolic racism was related to having a gun in the home and opposition to gun control policies in US whites. The findings help explain US whites’ paradoxical attitudes towards gun ownership and gun control. Such attitudes may adversely influence US gun control policy debates and decisions.

## Introduction

Several mass shootings in 2012 (e.g., Sandy Hook Elementary School, Connecticut; Aurora, Colorado) reignited gun-control and firearm ownership debates in the United States (US). The public health importance of gun reform in the US is clear and should not need such tragedies for policy change. In 2011, there were 32,163 firearm-related deaths in the US, with 11,101 homicides (69.5% of all homicides), and 19,776 suicides (51.6% of all suicides) [1]. Rates of firearm homicides in the US (3.6 per 100,000) are over 7-fold of those in similar nations (e.g., Canada, 0.5; United Kingdom, 0.1; Australia, 0.1) [2]. Blacks are disproportionately represented in US firearm homicides (14.6 per 100,000), and would benefit most from improved gun controls [1]. Opposition to gun control is considerably stronger in whites than blacks [3], with whites also reporting twice the rate of personal gun ownership and having a gun in the home, than is reported by blacks [4]. Proponents of gun-ownership rights cite self-protection and safety as their primary argument for owning guns and resisting gun reform [4], [5]. This is paradoxical, as whites, and particularly white males, are considerably more likely to commit suicide with firearms (7.3 and 12.9 per 100,000, respectively), than die from a firearm homicide (1.9 per 100,000) [1]. Indeed, US research found that having one or more guns in the home is related to a 2.7 and 4.8 fold increase in the risk of a member of that household dying from homicide or suicide, respectively [6], [7]. Given that gun controls have been shown to reduce suicides and homicides [8]–[10] arguments against gun reform based on self-defense/protection/safety are counterintuitive, and are inhibiting the adoption of appropriate policy to improve public health. As such, it is important for public health advocates, researchers, and policy makers to consider all explanations for opposition to gun reform in US whites. However, research on the reasons for opposition to gun control is sparse, in part because of restrictions on funding for research on gun control in the US [11], [12].

Stronger opposition to gun control by US whites has not always been the case. During the civil rights movement of the late 60 s, black activists exercised their right to carry loaded firearms in order to provide protection from police and extreme white factions [13]. The response from US whites was to demand stricter gun control. The Mulford Act was signed into law by Californian governor Ronald Reagan in 1967, and prohibited the carrying of loaded firearms in public [13]. The social landscape has changed considerably, and most recent data indicates a quite different view on gun control by whites, with 53% of whites wanting to protect the right to own guns, whereas only 24% of blacks do [14].

People’s stated reasons for owning guns and opposing gun-control legislation are likely complex; however, it has been suggested that sociocultural factors such as fear of black violence may be associated with gun ownership, and with opposition to gun controls [15], [16]. Similarly, negative attitudes towards blacks (i.e., racism), along with conservative and political ideologies, appear to be related to fear of black violence and crime [17]–[20]. What is not known, and accordingly is the focus of this study, is whether racism is associated with gun ownership and opposition to gun control. It has been found that racial stereotypes (e.g., that blacks are violent) are related to US whites’ fears of violence from blacks, and to their support for crime-related policy measures, such as building prisons, and the death penalty [19], [20]. Support for such policies is particularly pronounced in US whites who hold higher levels of racism [19]. Strong evidence also supports the notion that negative racial stereotypes and attitudes are related to people’s perceptions of threat from black gun-related violence [20]. Additionally, US research using measures of implicit race attitudes (e.g., Implicit Association Test; IAT) have shown a preference for whites over blacks [21] and appear to influence people’s political decisions, and even choices of medical procedures for blacks [22]–[24]. For instance, measures of explicit and implicit racism measures predicted opposition to Obama’s health reforms [23].

Most prominently, symbolic racism (racial resentment), an explicit but subtle form and measure of racism, has been found to be consistently related to peoples decisions regarding policies that may affect non-white US citizens. It is argued that symbolic racism supplanted old-fashioned or overt/blatant racism which had seen blacks as amoral and inferior, and was associated with open support for race inequality and segregation under ‘Jim Crow Laws’ [25]. Research following the US civil-rights movement suggested that anti-black racism and stereotyping, as assessed by blatant measures, had declined [26]. However, subsequent research revealed that people may merely be reluctant to express racism and negative stereotyping on these blatant measures in order to avoid appearing racist [27], [28]. This observation led to the conceptualization and measurement of more subtle measures of racism, such as, symbolic racism [25].

Symbolic racism is a belief structure underpinned by both anti-black affect and traditional values [29]. The anti-black affect (racism) component of symbolic racism is said to be established in pre-adult years through exposure to negative black stereotypes (e.g. blacks as dangerous, blacks are lazy), to the point that phenomena such as crime and physical violence have become typified as black phenomena [30]. The anti-black affect is not necessarily conscious or deliberative, but may be felt as fear, anger, unease, and hostility towards blacks [29], [31], [32]. The symbolic component reflects the abstract view of blacks as a collective rather than as individuals, as well as its basis in abstract white moralistic reasoning and traditions. Because symbolic racism represents an ingrained schema, individuals high in symbolic racism will react in a negative manner, often unconsciously, to issues perceived to involve a racial (i.e. black) component. Psychometric work shows that while symbolic racism has a small relationship with old-fashioned or blatant racism and stereotypes, only symbolic racism is associated with policy preferences related to race after controlling for conservative and political ideology and demographic characteristics (e.g., education, gender, age) [33].

Policies of which blacks or whites are the intended or obvious beneficiaries (e.g. affirmative action, school busing) should easily be perceived as involving a racial component. But other policies may also involve a perceived racial component merely because they concern an issue that is already understood by whites in racial (black) terms. Thus, symbolic racism has been linked to opposition to and support for a range of policies that whites consistently associate with blacks (e.g., welfare), even if it is not in the self-interest of whites to do so [22]–[25], [32]. This is also likely to explain the frequently observed correlations between symbolic racism and public opinion regarding a range of criminal justice policies (e.g. death penalty, mandatory sentences). There is substantial evidence that whites associate blacks with crime, and especially violent crime [19], [30]. The result of this conflation of race and crime is that whites high on symbolic racism will support policies that are perceived as being tough on crime and oppose policies that are considered lenient. Green and colleagues [34] have found a positive relationship between symbolic racism and punitive crime policies (i.e., death penalty, three strikes imprisonment), and negative correlation with policies that are intended to assist criminals (i.e., education of inmates, poverty reduction). And although conservative ideologies and racism are inherently related, symbolic racism makes a unique contribution to crime policy attitudes after accounting for other race-neutral factors (e.g., conservatism, crime victimization, crime news exposure, and socio-demographics) [34]. More generally, symbolic racism should also correlate with fear of crime and black violence, along with attitudes to policies that may reduce, or increase, perceived threat (e.g., gun ownership, gun control). Self-protection and physical safety (e.g., fear) are the most commonly cited reason for owning a gun and opposing gun control and blacks are overrepresented in the crime statistics and media portrayals of violent crime. Accordingly, people with higher symbolic racism may be more likely to own a gun and oppose gun control as a means of dealing (consciously or unconsciously) with abstract fears regarding blacks [19].

Given the importance of guns and gun-control to US public health, and the urgent need for appropriate policy to reduce gun-related harms, it is vital to examine the psychological and sociocultural reasons for the paradoxical attitudes of many US citizens and politicians to gun-control. US whites have twice the rate of gun ownership of blacks, oppose gun control to much greater extent than blacks, but are considerably more likely to kill themselves with those guns, than be killed by others or blacks. While the literature suggests that racism in whites shapes fear of black violence and support for policies that disadvantage blacks, no research has examined whether racism is related to gun ownership and attitudes to gun-control in US whites. This study investigated whether racism is related to gun ownership and opposition to gun control in US whites. We hypothesized that, after accounting for known confounders (i.e., age, gender, education, income, location, conservatism, political identification, anti-government sentiment), anti-black racism would be associated with having a gun in the home, and opposition to gun controls.

## Methods

The most recent data from the American National Election Study (ANES) [35] was used to test the hypothesis. The ANES panel study is the leading large-scale psychological and socio-political attitudes survey in the US, measuring various constructs and attitudes in monthly waves from a representative probability sample of US voters. Explanatory variables, including demographic details (i.e., age, gender, education, income, location: southern vs. other), anti-government sentiment, measures of conservatism (e.g., liberal versus conservative ideology), party identification (e.g., Republican versus Democrat leanings), symbolic racism, belief in a black violent stereotype, and implicit racism (i.e., race IAT), were accessed for US whites. Outcome measures were: having a gun in the home, opposition to policies banning handguns in the home, and support for permits to carry concealed handguns.

Potential participants for the ANES were contacted via telephone using random-digit-dialling and requested to complete an online survey each month from January 2008 to September 2009. Respondents were paid $10 a month for participation and those without internet access were provided with internet service for the duration of the study. The current study drew on data from several waves of the ANES survey. To counter the impact of participant drop-out and non-response on the representativeness of the sample examined in the current study we applied ANES generated weights as recommended (i.e. wave 20 post-election weight) [35]. The comprehensive ANES panel study demographics, data, materials and methods are freely available online at (http://www.electionstudies.org/).

## Measures

As part of the ANES, participants provided comprehensive information about the demographic composition of their household alongside their own background characteristics. Participants’ highest level of educational attainment was grouped into five categories: less than high school diploma, high school diploma, some college but no bachelor’s degree, bachelor’s degree, and graduate degree. This variable was scored from 1 = less than high school diploma, to 5 = graduate degree. Household income in the last year was reported by all participants. Participants were instructed to include their own pre-tax income and the income of all other household members from all sources (e.g. wages, tips, interest on savings, child support, Social Security). Nineteen income bands were used ranging from 1 = <$5,000 per annum to 19 = ≥$175,000 or more per annum. Consistent with previous research [36], education and income where dummy coded into five and four categories for analysis, respectively, rather than being treated as linear variables.

### Racism

Measures of two key types of racism against blacks were taken from the ANES for analyses: symbolic racism and implicit racial attitudes. Additionally, a single item from wave 20 of ANES was used to assess whether participants held the stereotype that blacks are violent. Participants responded to the item “How well does the word ‘violent’ describe most blacks?” using five response categories ranging from 1 = “extremely well”, to 5 = “not at all well” (i.e. extremely well, very well, moderately well, slightly well, or not at all well). The item was coded so that a response of extremely well or very well, indicated endorsement of the black violent stereotype (coded 1), with other responses coded as 0, did not endorse stereotype blacks are violent.

In wave 20 of the ANES, participants were asked to respond to a four-item scale drawn from the Symbolic Racism Scale [37]. Specifically, participants indicated the extent to which they agree (1 = agree strongly to 5 = disagree strongly) with statements such as “Generations of slavery and discrimination have created conditions that make it difficult for blacks to work their way out of the lower class” (reverse scored). Scores on the four items were coded so that high scores are indicative of elevated levels of symbolic racism. A test of the reliability of the scale showed the four items corresponded closely with each other as indicated by a Cronbach’s alpha level of 0.8 and the emergence of a single factor from exploratory factor analysis of the scale. We utilized the average score across the four items to produce a scale ranging 1 = lowest symbolic racism score, to 5 = highest symbolic racism score.

The Implicit Association Test (IAT) is commonly used in experimental psychology to gauge implicit bias. A brief race (anti-black) IAT was included in wave 19 of the ANES to assess the extent to which participants demonstrated black-white racial bias. The theoretical background, instructions, and methodology for the race IAT have been well described elsewhere [21], [22]. Briefly, the race IAT was administered online, requiring participants to rapidly associate pictures of white and black faces with positively- and negatively-valenced words. Participants were asked to press the key “P” for white faces and for positive words and “Q” for any other stimulus. Alternatively, they were asked to press “P” for black faces or positive words and “Q” for other stimuli. The test consisted of 84 stimuli, two practice runs (14 sets of stimuli each) and two data collection blocks (28 sets of stimuli each). Response latencies across blocks were analysed to produce an effect size coefficient or *D* score. This score is coded so that positive scores indicate an unconscious preference for whites over blacks.

### Conservatism, Anti-Government Sentiment, and Political Party Identification

Conservatism (ideological self-placement) was derived from four items assessing self-descriptions of liberal versus conservative leanings, and strength thereof. The four items were asked in wave 11 of the ANES. Participants were firstly asked “When it comes to politics, would you describe yourself as liberal, conservative, or neither liberal nor conservative?”. The extent to which participants considered themselves to be liberal or conservative was then gauged with a further question: “Would you call yourself very liberal or somewhat liberal? Would you call yourself very conservative or somewhat conservative?”. Those who rated themselves as “neither liberal nor conservative” were requested to indicate: “Do you think of yourself as closer to liberals, or conservatives, or neither of these?”. We combined all ratings on these four items to produce a score ranging from 1 to 7 (1 = extremely liberal, 4 = moderate, 7 = extremely conservative).

To better capture conservative values and associated views regarding government infringement on personal rights, we included a measure of anti-government sentiment. Participants responded with either a yes, immediate threat; or no, does not (yes responses coded as 1, no as 0) to the item ‘Do you think the federal government has become so large and powerful that it poses an immediate threat to the rights and freedoms of ordinary citizens, or not?’.

Party identification, and the strength of this identification, was derived (wave 19) from the same process using four component questions assessing whether participants identified themselves as Republicans, Democrats, or Independents. This process yielded a score ranging from 1 to 7 (1 = strong Democrat, 4 = independent, 7 = strong Republican).

### Gun Ownership

Questions relating to household gun ownership were included in wave 19 of ANES. Participants were firstly asked if any person in the household owned any type of gun. Specifically, participants were asked: “Do you or does any other member of your household own a handgun, rifle, shotgun, or any other kind of firearm, or does no one in your household own a firearm?”. Subsequently, participants were asked: “Do you happen to have in your home or garage any guns or revolvers?”. This second question functioned largely to corroborate responses to the initial question, but also established the participant’s personal ownership of the reported gun in the home. For analyses, a yes response to either item was coded as a 1, no responses were coded as 0.

### Opinions on Gun Control

Participants were asked two questions regarding their views on two potential gun control policies in wave 13 of the ANES panel study. Participants were firstly asked: “Do you favor, oppose, or neither favor nor oppose making it illegal for anyone to keep a handgun at home?”. Next they were asked: “Do you favor, oppose, or neither favor nor oppose giving permits to allow any adult to carry a concealed handgun if they have never been convicted of committing a crime and they have passed a test showing that they know how to use the gun safely?”. To produce a clear index of whether the participant is opposed to gun control, we coded responses to the first question so that 1 = definite opposition to making it illegal to keep a handgun at home, and 0 = other responses. The item assessing support for a permit to carry a concealed handgun was reverse coded, so that “favor” for permits to have concealed handguns was coded as 1, which in effect represents opposition to gun control. Other responses were coded as 0, indicating non-support for concealed handguns.

### Statistical Analysis

Multivariate logistic regression was used to examine relationships between explanatory variables, and gun-related outcomes. Odds ratios (OR) are reported with 95% confidence intervals (CI) for univariate and multivariate relationships with the outcome variables (see Tables 1–3) based on Taylor linearized standard errors. Explanatory variables were entered simultaneously in models, with the exception of having a gun in the home. Because participants reporting gun ownership will quite logically be against measures that involve giving up their guns, and ownership is hypothesised to be related to racism, we entered the variable ‘have a gun in the home’ in a second step for models examining opposition to gun control (Tables 2 and 3). Spearman’s correlation coefficients between all variables were calculated along with descriptives (see Tables 4 and 5, respectively).

**Table 1:** Univariate and multivariate relationships from logistic regressions are displayed for having a gun in the home among US whites.

		Gun in home	
		Univariate	Multivariate^a^
Explanatory Variables		OR (95% CI)	OR (95% CI)
**Age**		1.00 (1.00, 1.01)	1.01 (1.00, 1.02)
**Education**	No High School Diploma	1.28 (0.60, 2.73)	Reference
	High School Diploma	1.25 (0.89, 1.77)	0.64 (0.27, 1.55)
	Some college, no bachelor’s degree	1.26 (0.95, 1.67)	0.62 (0.26, 1.47)
	Bachelor’s degree	0.69** (0.51, 0.93)	0.46 (0.19, 1.11)
	Graduate degree	0.55*** (0.41, 0.75)	0.39* (0.16, 0.96)
**Income**	Under $20,000	0.43** (0.23, 0.81)	Reference
	$20,000 to $49,999	1.16 (0.85, 1.59)	2.05* (1.03, 4.09)
	$50,000 to $99,999	1.10 (0.82, 1.47)	2.24* (1.13, 4.43)
	$100,000 or more	0.92 (0.66, 1.27)	2.04 (0.96, 4.34)
**Male**		1.24 (0.93, 1.64)	1.06 (0.77, 1.45)
**Southern** (yes = 1, no = 0)		1.44* (1.05, 1.96)	1.15 (0.82, 1.62)
**Conservatism**		1.26*** (1.17, 1.37)	1.06 (0.94, 1.18)
**Anti-Government Sentiment** (yes = 1, no = 0)		1.97*** (1.44, 2.69)	1.38 (0.98, 1.96)
**Party Identification**		1.22*** (1.14, 1.30)	1.10 (0.99, 1.21)
**Black Violent Stereotype**		1.15 (0.69, 1.93)	0.90 (0.52, 1.54)
**Implicit Racism**		1.32 (0.97, 1.79)	1.16 (0.84, 1.61)
**Symbolic Racism**		1.86*** (1.57, 2.20)	1.50*** (1.22, 1.84)

*** p<0.001;**p<0.01;*p<0.05;

a Adjusting for all other variables in the model. All VIF-values are below 2.00.

Link-tests indicated that multivariate model are correctly specified (t-statistic was 0.39). F-adjusted mean residual tests for goodness-of-fit for the dependent variable indicated a good fit (p value 0.59).

**Table 2:** Univariate and multivariate relationships from logistic regressions are displayed for opposition to a handgun ban in home among US whites.

		Opposition to handgun ban in home		
		Univariate	Multivariate^a^	Multivariate^b^
Explanatory Variables		OR (95% CI)	OR (95% CI)	OR (95% CI)
**Age**		1.00 (0.99, 1.01)	1.00 (0.99, 1.02)	1.00 (0.99, 1.01)
**Education**	No High School Diploma	1.61 (0.74, 3.48)	Reference	Reference
	High School Diploma	0.80 (0.56, 1.15)	0.49 (0.19, 1.23)	0.53 (0.20, 1.41)
	Some college, no bachelor’s degree	1.51**(1.11, 2.05)	0.78 (0.32, 1.90)	0.86 (0.33, 2.22)
	Bachelor’s degree	1.08 (0.79, 1.49)	0.72 (0.28, 1.83)	0.86 (0.32, 2.27)
	Graduate degree	0.74 (0.54, 1.00)	0.57(0.22, 1.47)	0.72 (0.27, 1.96)
**Income**	Under $20,000	0.68(0.37, 1.26)	Reference	Reference
	$20,000 to $49,999	0.82 (0.59, 1.14)	1.04 (0.50, 2.14)	0.88 (0.41, 1.86)
	$50,000 to $99,999	1.18 (0.87, 1.61)	1.25 (0.61, 2.57)	1.05 (0.50, 2.21)
	$100,000 or more	1.18 (0.83, 1.67)	1.16 (0.54, 2.50)	0.98 (0.45, 2.16)
**Male**		2.11***(1.54, 2.87)	1.72**(1.21, 2.44)	1.69*(1.17, 2.44)
**Southern** (yes = 1, no = 0)		2.05***(1.44, 2.92)	1.72**(1.17, 2.54)	1.67*(1.11, 2.54)
**Conservatism**		1.40*** (1.29, 1.53)	1.15*(1.02, 1.30)	1.14*(1.01, 1.30)
**Anti-Government Sentiment** (yes = 1,no = 0)		2.47***(1.80, 3.40)	1.78**(1.23, 2.58)	1.68**(1.15, 2.45)
**Party Identification**		1.32***(1.22, 1.42)	1.15**(1.04, 1.28)	1.13*(1.02, 1.26)
**Black Violent Stereotype**		1.38 (0.76, 2.53)	1.37 (0.74, 1.54)	1.46 (0.78, 2.76)
**Implicit Racism**		1.07 (0.77, 1.50)	1.01 (0.69, 1.47)	0.98 (0.66, 1.45)
**Symbolic Racism**		1.70***(1.43, 2.02)	1.27*(1.03, 1.58)	1.17 (0.94, 1.46)
**Gun in the home** (yes = 1, no = 0)		3.38***(2.77, 5.45)	–	2.84***(1.95, 4.14)

*** p<0.001;**p<0.01;*p<0.05;

a Adjusting for all other variables in the model except having gun in home.

b Adjusting for all other variables in the model including having gun in home. All VIF-values are below 2.02. Link-tests indicate that multivariate model was correctly specified (t-statistic.69). The p value for the F-adjusted mean residual test for goodness-of-fit was (0.18).

**Table 3:** Univariate and multivariate relationships from logistic regressions are displayed for support for permits to carry concealed handguns among US whites.

		Support permit for concealed handguns		
		Univariate	Multivariate^a^	Multivariate^b^
Explanatory Variables		OR (95% CI)	OR (95% CI)	OR (95% CI)
**Age**		1.00 (0.99, 1.01)	1.00 (0.99, 1.01)	1.00 (0.99, 1.01)
**Education**	No High School Diploma	1.61 (0.74, 3.48)	Reference	Reference
	High School Diploma	1.10 (0.56, 1.15)	0.50 (0.21, 1.19)	0.54 (0.23, 1.29)
	Some college, no bachelor’s degree	1.13 (0.86, 1.50)	0.47 (0.20, 1.08)	0.50 (0.22, 1.13)
	Bachelor’s degree	0.86 (0.64, 1.15)	0.43 (0.18, 1.04)	0.49 (0.21, 1.15)
	Graduate degree	0.54***(0.40, 0.73)	0.32*(0.13, 0.77)	0.38*(0.16, 0.91)
**Income**	Under $20,000	0.68(0.37, 1.26)	Reference	Reference
	$20,000 to $49,999	1.19 (0.87, 1.63)	1.18 (0.61, 2.27)	1.01 (0.51, 2.02)
	$50,000 to $99,999	0.96 (0.73, 1.28)	1.08 (0.57, 2.07)	0.92 (0.46, 1.81)
	$100,000 or more	0.94 (0.68, 1.30)	1.12 (0.55, 2.30)	0.95 (0.46, 1.96)
**Male**		2.29***(1.72, 3.05)	2.01***(1.45, 2.79)	1.99***(1.43, 2.77)
**Southern** (yes = 1, no = 0)		1.77***(1.30, 2.42)	1.46*(1.03, 2.06)	1.41(0.97, 2.05)
**Conservatism**		1.37***(1.26, 1.50)	1.14*(1.01, 1.29)	1.14*(1.01, 1.29)
**Anti-Government Sentiment** (yes = 1,no = 0)		2.47***(1.80, 3.40)	1.89**(1.30, 2.73)	1.81*(1.24, 2.66)
**Party Identification**		1.29***(1.20, 1.38)	1.14*(1.03, 1.27)	1.13*(1.01, 1.25)
**Black Violent Stereotype**		1.75*(1.03, 2.95)	1.22 (0.71, 2.09)	1.27 (0.72, 2.25)
**Implicit Racism**		1.10 (0.81, 1.50)	0.95 (0.66, 1.39)	0.94 (0.64, 1.36)
**Symbolic Racism**		1.84***(1.57, 2.17)	1.38**(1.13, 1.70)	1.28*(0.94, 1.46)
**Gun in the home** (yes = 1, no = 0)		3.29***(2.44, 4.42)	–	2.50***(1.78, 3.51)

*** p<0.001;**p<0.01;*p<0.05;

a Adjusting for all other variables in the model, except having a gun in home.

b Adjusting for all other variables in the model including having a gun in home. VIF-values were below 2.04. Link-tests indicate that multivariate model was (t-statistic 0.04). F-adjusted mean residual tests for goodness-of-fit p value 0.04.

**Table 4:** Spearman’s correlation coefficients for bivariate relationship between all variables.

Variables	1	2	3	4	5	6	7	8	9	10	11	12	13	14	15	16	17	18	19	20
**1. Gun in the home**																				
**2. Oppose banning guns in the home**	.35***																			
**3. Support for permit concealed weapon**	.33***	.47***																		
**4. Age**	.05	−.01	−.05*																	
**5. No high school diploma**	.04	.03	.05*	.01																
**6. High school diploma**	.05	−.03	.04	.05	−.06*															
**7. Some college, no bachelor**’**s degree**	.10**	.10**	.10**	−.01	−.11**	−.32***														
**8. Bachelor**’**s degree**	−.04	−.01	−.05*	−.09**	−.09**	−.25***	−.45***													
**9. Graduate degree**	−.14**	−.09**	−.11**	.08**	−.07**	−.21***	−.38***	−.30***												
**10. Under $20,000**	−.05	−.03	−.04	.08**	.06*	.12**	.03	−.08**	−.08**											
**11. $20,000 to $49,999**	−.01	−.04	.02	.12**	.06*	.17**	.06*	−.11**	−.13**	−.15**										
**12. $50,000 to $99,999**	.07**	.05*	.03	−.06*	−.04	−.05	.05	.02	−.03	−.20**	−.51***									
**13. $100,000 or more**	−.04	−.01	−.03	−.09**	−.05	−.19**	−.14**	.13**	.20***	−.15**	−.37***	−.49***								
**14. Male**	.11**	.18**	.18**	.07*	−.02	−.07*	−.04	.06*	.05	−.08*	−.11**	.09*	.05*							
**15. Southern**	.09**	.11**	.11**	.06*	.01	.01	.02	.01	−.04	−.02	.03	−.03	.00	.04						
**16. Conservatism**	.26***	.31***	.33***	.05	−.01	.07*	.11**	−.06*	−.13**	−.06*	.02	.01	−.00	.13**	.11**					
**17. Anti**−**government sentiment**	.15**	.20***	.20***	.04	.02	−.00	.05*	−.02	−.05	.01	.01	.03	−.05	.15**	.03	.20***				
**18. Party identification**	.22***	.30***	.30***	−.03	−.03	.01	.11**	−.02	−.10**	−.09**	−.00	.02	.03	.08*	.11**	.70***	.16**			
**19. Implicit racism**	.06*	.04	.05	.05	.07*	.05	.02	−.04	−.05	−.01	.05*	−.00	−.04	−.02	.02	.06*	.01	.06*		
**20. Black violent stereotype**	.08*	.07*	.09**	−.04	.03	.07*	.08**	−.08**	−.08**	−.03	.08**	.02	−.08**	.04	.02	.06*	.06*	.04	.09**	
**21. Symbolic racism**	.26***	.29***	.31***	−.08*	.00	.10**	.19***	−.08**	−.23***	−.04	.03	.02	−.03	.10**	.12**	.46***	.22***	.41***	.15**	.24***

Significant two-tailed relationships are indicated ***p<0.001; **p<0.01; *p<0.05.

**Table 5:** Weighted means and 95% confidence intervals for all variables.

	Mean	Lower CI	Upper CI	Range	N†
**Gun in home (1 = yes, 0 = no)**	0.52	0.49	0.56	0, 1	1354
**Opposition to handgun ban in home (1 = yes, 0 = no)**	0.66	0.63	0.70	0, 1	1370
**Support permit for concealed handguns (1 = yes, 0 = no)**	0.52	0.49	0.56	0, 1	1370
**Age**	49.35	47.97	50.73	18–90	1370
**Education**	2.94	2.86	3.03	1–5	1370
**No High School Diploma**	0.08	0.05	0.11	0, 1	1370
**High School Diploma**	0.30	0.27	0.34	0, 1	1370
**Some college, no bachelor**’**s degree**	0.31	0.28	0.34	0, 1	1370
**Bachelor**’**s degree**	0.21	0.18	0.23	0, 1	1370
**Graduate degree**	0.10	0.09	0.12	0, 1	1370
**Income**	12.51	12.27	12.75	1–19	1368
**Under $20,000**	0.05	0.04	0.07	0, 1	1368
**$20,000 to $49,999**	0.31	0.27	0.34	0, 1	1368
**$50,000 to $99,999**	0.43	0.39	0.46	0, 1	1368
**$100,000 or more**	0.21	0.19	0.24	0, 1	1368
**Sex (1 = male, 0 = female)**	0.49	0.45	0.52	0, 1	1370
**Conservatism**	4.56	4.43	4.68	1–7	1370
**South (1 = yes, 0 = no)**	0.32	0.28	0.35	0, 1	1370
**Anti-Government Sentiment**	0.70	0.66	0.73	0, 1	1370
**Party identification**	4.16	4.01	4.31	1–7	1370
**Black violent stereotype** *	0.10 (2.1)	0.08 (2.14)	0.13 (2.29)	0, 1 (1–5)	1360
**Implicit racism**	0.17	0.13	0.20	−2–2	1287
**Symbolic racism**	3.48	3.42	3.55	1–5	1370

Unequal N’s result from lower non-assessment rates for a measure in a specific ANES wave or from missing.

The means and confidence intervals are calculated using svy: mean procedure and are based on the white subsample.

† N reflects size of the population of whites after weighting. Range refers to the theoretical range.

* In brackets is the mean score for 5-point scales for black violent stereotype.

## Results

Just over half (52%) of the sample had a gun in the home, 66% opposed bans on handguns in the home, and 52% reported support for permits to carry a concealed handgun. Participants reported being slightly more conservative than liberal, and more Republican than Democratic leaning. Mean scores for symbolic racism, and to a lesser extent the race IAT, indicated anti-black sentiment; however, participants had mean scores considerably below the midpoint of scoring for the stereotype that ‘blacks are violent’. Table 5 displays full weighted descriptives.

After adjusting for all explanatory variables in the model, symbolic racism was significantly related to having a gun in the home. Specifically, for each 1 point increase in symbolic racism, there was a 50% greater odds of having a gun in the home (see Table 1), and there was a 28% increase in the odds of supporting permits to carry concealed handguns (see Table 3). The relationship between symbolic racism and opposing a ban on guns in the home (27% increase in odds), was reduced (17% increase in odds) and became non-significant when the outcome ‘having a gun in the home’ was entered in the model (see Table 2). This is unsurprising as, in effect, opposition to gun control policy is conflated with having a gun already, and reflects self-interest [38]. Thus the gun ownership variable mediated the relationship between symbolic racism and opposition to a ban on handguns in the home. It is noteworthy that symbolic racism still maintained its significant relationship with support for permits to carry concealed handguns in the presence of having a gun in the home.

Conservative ideology was also significantly related to stronger support for permits to carry concealed handguns after adjusting for other explanatory variables. Similarly, stronger republican identification, being from a southern state, and anti-government sentiment were associated with opposition to gun-control policies, but not with having a gun in the home. With the exception of sex, and to a much lesser extent education, demographic variables were not related to having a gun in the home or opposition to gun controls. Although sex was unrelated to having a gun in the home, there were greater odds of males being opposed to banning handguns in the home, and being supportive of permits to carry concealed handguns, than for females. This result is consistent with other US data showing that white males display the most opposition to gun control, and greater support for liberalisation of gun laws [3]. Higher education levels were associated with lower odds of having a gun in the home, but not with the gun control outcomes. This finding mirrors national data on gun ownership and support for gun control policies [3], which also shows a poor and mixed relationship between income and age, and gun ownership.

In correlation analyses, greater race IAT scores were weakly associated with greater symbolic racism scores, and with the black violent stereotype. Higher IAT scores were not related to gun ownership and gun control in full models. Higher scores on black violent stereotyping were not related to any of the gun-related outcomes; the univariate relationship between black violent stereotyping and greater support for concealed handgun permits was explained by other variables.

## Discussion

Opposition to gun control in US whites is somewhat paradoxical given the statistics on gun-related deaths, and such opposition may be undermining the public health of all US citizens. This study examined for the first time whether racism is related to gun ownership and the opposition to gun control in US whites. The results support the hypothesis by showing that greater symbolic racism is related to increased odds of having a gun in the home and greater opposition to gun control, after accounting for all other explanatory variables.

It is particularly noteworthy that the relationship between symbolic racism and the gun-related outcomes was maintained in the presence of conservative ideologies, political affiliation, opposition to government control, and being from a southern state, which are otherwise strong predictors of gun ownership and opposition to gun reform. Contrary to research showing associations between implicit racism and policy decision making [23], we did not find implicit racism to be significantly related to gun related outcomes after accounting for other variables. Similarly, the small correlations between the stereotype that most blacks being violent and gun outcomes were not significant after accounting for all other variables.

There are several possible reasons for the absence of multivariate associations between the stereotype of blacks as violent and race-IAT, and gun outcomes. There is considerable debate in the field regards the validity and predictive qualities of implicit measures with critical reviews and reanalyses showing weak or no association between implicit and explicit measures, and outcomes [39], [40]. Others demonstrate that non-attitudinal factors, such as, stimuli familiarity, cognitive ability, and fear of appearing racist also account for individual differences in IAT scores, that may in turn affect associations with outcome variables [39]–[43]. The implicit association test is also a conceptually difficult task for some to learn, and particularly the brief race-IAT used in the ANES which restricts training on this computerized measure [41]. Given the mean *D* score for the ANES race-IAT (.17) is more than twice as small as from any other studies, including one in medical doctors [44], it is also possible that participants may not have completed this complex computerized task correctly. Other authors have noted this problem with the ANES race-IAT data [45].

There are two plausible reasons for the blacks as violent stereotype not accounting for significant variance in multivariate models. First, the stereotype appears to be subsumed by symbolic racism. Table 4 shows that the black violent stereotype has its strongest relationship with symbolic racism (r = .24), and only weak relationships with other variables (rs = .06–.09). Thus, the association between the black violent stereotype and gun outcomes may be explained through its association with symbolic racism which captures negative affect towards blacks (e.g., fear, unease, hostility). Alternatively, because the black violent stereotype is a quite blatant measure, participants may have been reluctant to endorse a clearly negative view of blacks in order to avoid appearing racist. In support of this notion, only 10% of participants strongly endorsed the statement that most blacks could be described as violent, with a mean score of 2.2 on the 5-point scale, compared to a mean score of 3.5 for symbolic racism on a 5-point scale.

There are potential limitations that should be noted. The item assessing having a gun in the home does not establish that the respondent is the owner or user of that gun. This observation is born out in the absence of a sex difference to this question. Males typically have a higher rate of gun ownership than females [3]. Similarly, the gun control policy items do not assess opposition/support for assault weapons, which has been a particular focus of attention during recent gun debates in the US. Nonetheless, symbolic racism might also, quite reasonably, be related to opposition to broader gun control measures (banning assault weapons, and gun clips containing more than 10 rounds), which may or may not be effective in reducing firearms related deaths. However, although the ANES only asked participants whether there was a gun in the home, best available evidence suggests that merely having a gun in the home is associated with a marked increase in the odds of one of the members of that home dying from suicide or homicide [6], [7].

Another potential limitation is the focus on white US adults as it is possible that other US racial groups may display similar pattern of results. However, given that whites oppose gun reforms to a considerably greater extent than do blacks, or indeed any other non-white racial group, that whites are also the single largest (>70%) ethnic grouping in the US, and that symbolic racism in whites is related to numerous outcomes, the focus of the study on whites seems appropriate [3]. Indeed, in a sub-analysis of the black sample from the ANES panel study, we found that none of the variables reported in models for white participants were significantly related to any of the gun-related outcomes for blacks. Finally, the correlational nature of the study clearly prohibits causal inferences. While a view that racism underpins gun-related attitudes is plausible and supported by evidence on other race-related policy decisions [18], [23], it could be argued that there are other plausible but unmeasured variables that could explain the pattern of relationships we find here. Similarly, simply owning a firearm may lead whites to develop more negative attitudes towards blacks. There is some experimental research showing that participants who have recently held a firearm produce enhanced salivary testosterone levels and display increased aggression toward others [46]. Causality aside, greater control of firearms is the most logical direction for public health policy.

Notwithstanding these limitations, the results indicate that symbolic racism is associated with gun-related attitudes and behaviours in US whites. The statistics on firearm-related suicides and homicides in the US might reasonably be expected to convince US citizens that action on reducing gun ownership and use would be beneficial to their health. Yet, US whites oppose strong gun reform more than all other racial groups, despite a much greater likelihood that whites will kill themselves with their guns (suicide), than be killed by someone else [1]. Black-on-black homicide rates would benefit most from gun reform, and, quite logically, blacks support these reforms even if whites do not [3], [47]. Symbolic racism appears to play a role in explaining gun ownership and paradoxical attitudes to gun control in US whites. In other words, despite certain policy changes potentially benefitting whites, anti-black prejudice leads people to oppose their implementation. This finding is consistent with previous research showing that symbolic racism is associated with opposition to US policies that may benefit blacks, and support for policies that disadvantage blacks, and critically, goes beyond what is explained by other important confounders.

Gun-related deaths in the US are a significant public health concern, representing a leading cause of death, and are particularly prevalent from ages 15–54. Attitudes towards guns in many US whites appear to be influenced, like other policy preferences, by illogical racial biases. The present results suggest that gun control policies may need to be implemented independent of public opinion. The implementation of initially unpopular public health initiatives has proven effective for other public health threats (e.g., tobacco taxation, bans on smoking in public places, seatbelt use) that initially did not have widespread public and political support, but have eventually proven popular and have led to changes in attitudes [48], [49].

There remains considerable resistance in the US to even cursory gun controls, and the reasons for owning a gun and opposing gun reform (i.e., self-protection, safety, fear of crime) [4], [5], are not supported by the evidence on gun-related harms. Clearly, other motives and attitudes must be driving such paradoxical views on guns. Future research needs to examine other less obvious, yet influential, sociocultural and psychological influences on gun ownership and control, as this evidence is sparse. Evidence on the psychological and sociocultural drivers of gun ownership and resistance to strong controls will in turn help inform educational campaigns (e.g., social marketing) that may aid public acceptance of appropriate policies in the interest of the US public’s health, and/or allow policy makers to implement good public health policy. The reinstatement of funding for research on gun control in the US should assist in these research endeavours.
